# Effectiveness of fibrin glue in skin graft survival: A systematic review and meta-analysis

**DOI:** 10.1016/j.amsu.2020.06.006

**Published:** 2020-06-12

**Authors:** Ekta Paw, Venkat Vangaveti, Mark Zonta, Clare Heal, Ronny Gunnarsson

**Affiliations:** a College of Medicine and Dentistry, James Cook University, Townsville, Australia; b Townsville University Hospital, Townsville, Australia; c Primary Health Care, School of Public Health and Community Medicine, Institute of Medicine, Sahlgrenska Academy, University of Gothenburg, Gothenburg, Sweden

**Keywords:** Fibrin, Skin graft, Skin transplant

## Abstract

**Background:**

The objective of this study is to assess the current literature on the effectiveness of fibrin glue on survival of skin grafts. Fibrin glue is a possible alternative to secure skin grafts instead of traditional methods (i.e. sutures or staples).

**Methods:**

Data Sources: MEDLINE, Scopus, Embase, Informit, CINAHL and the Cochrane Central Register of Controlled Trials, no limit on the earliest date of publication.

**Study eligibility criteria:**

Randomised, non-randomised controlled trials and cohort studies.

**Participants:**

and Interventions: Participants were patients with skin grafting/skin transplantation. The intervention was fibrin glue in any form (bovine, human pooled plasma or autologous) and comparator any form of affixing skin grafts (e.g. sutures or staples).

Study Appraisal and Synthesis Methods: Studies were appraised using the Cochrane risk of bias tool and assessed for clinical heterogeneity. Effect sizes were calculated and illustrated with forest plots.

**Results:**

190 publications were narrowed to 15 relevant publications, of which eight were pooled in meta-analysis. The outcomes examined were: graft survival by percentage; graft survival reported as events; post-operative incidence of haematoma or seroma; pain reported after dressing changes via a visual analogue scale; length of stay in days (Glass's delta 2 was 0.48 95% CI 0.09, 0.97); and surgical time in minutes. Only length of stay showed a difference between groups and it favoured fibrin glue.

**Conclusions:**

While there may be benefits to the use of fibrin glue in skin graft patients, it is difficult to conclude this from the current evidence. Limitations were significant heterogeneity in outcomes measured and exclusion off non-English papers.

## Highlights

• Largest systematic review of fibrin glue in skin grafts to date.
• Both clinical and patient reported outcomes important in assessing skin grafts.
• Significant Improvement in length of stay for skin graft patients with fibrin glue.
• Meta-Analysis of graft survival, pain and operative time for skin graft patients who had fibrin glue.

## 1 Introduction

### 1.1 Rationale

Fibrin glue, composed of thrombin, factor XIII, calcium and fibrinogen, was initially developed for use as a haemostatic agent. When introduced to a wound it mimics the final events in the clotting cascade, turning fibrinogen into fibrin [[1], [2], [3]]. When applied between the skin graft and the wound it produces a biological adherent over the entire surface of the graft, as opposed to the traditional method of fixation (i.e. sutures or staples) where adherence is only secured at the edges of the graft. Originally the source of fibrin was centrifuged patient plasma. Now commercial preparations of high concentrations of fibrin are now available and can be used as adhesives [4]. There are a few main subtypes of fibrin glue used in practice: autologous glue, where the patient's own plasma is processed to maintain only the fibrin rich aspects; homologous glue where plasma is taken from a number of volunteers and processed to become fibrin rich; and bovine glue where fibrin proteins are extracted from bovine plasma [[5], [6], [7]].

Fibrin glue has been investigated for use in skin grafts for burns and compared to staples/sutures in terms of wound closure [8]. Skin graft survival is dependent on vascularisation of the graft which usually begins after 2–3 days. During this period poor adherence or haematoma/seroma formation can disrupt further vascularisation and lead to graft failure [9]. Specifically, because fibrin glue adheres the entire surface of the graft to the wound, it can reduce the formation of haematoma or seroma immediately post operatively [9]. Studies have also shown less requirement for dressings to ensure close adherence of the graft, better haemostasis and less contraction of scar tissue for various uses of skin grafts [7,10,11]. Issues with the graft recipient site such as poor vascularity, infection and inflammation can also lead to graft failure [9]. Fibrin glue has been investigated for use in other difficult to graft situations such as infected sites and over joint surfaces [[12], [13], [14], [15], [16], [17]]. It is possible that fibrin glue can increase graft survival in patients who have vascular issues as it has been shown that increased fibrin decreases likelihood of graft failure and can induce angiogenesis [18].

Queensland, Australia has the highest rate of skin cancer in the world [19]. Moreover, the Australian Institute of Health and Welfare, also notes that melanoma related hospitalisations have risen 63% in the last decade [19]. Admitted patients are usually those needing large excisions to obtain adequate surgical margins and may require skin grafts to close their defects [20]. Patients with skin cancer are more likely to be elderly and with comorbidities and therefore carry an increased risk of graft failure, as opposed to burns patients where children are highly represented [21,22].

Sixty-five review papers were found, however only two of these were systematic and both only examined burns patients [23,24]. There is a need for a review that is much broader in scope, and has been conducted more extensively than what is currently published. Given this context, the aim of this review is to broadly study the literature around fibrin glue and its potential applicability to skin cancer patients.

### 1.2 Objectives

The objective for this systematic review is to ascertain to what extent the current published literature supports the use of fibrin glue in skin grafts, and the quality of that literature. This review will also question whether this literature can be applied to the population of patients requiring grafting for skin cancer, as published results suggest that much of the literature relating to skin grafts pertains specifically to burns patients.

## 2 Methods

### 2.1 Protocol and registration

The systematic review protocol was developed in accordance with PRISMA guidelines and the study protocol was published prospectively in the PROSPERO international prospective register of systematic reviews (CRD42018088263).

### 2.2 Eligibility criteria

Prospective studies, including randomised controlled trials, controlled trials and cohort studies with a comparator group were eligible. The eligibility was extended beyond randomised controlled trials due to the small number of published papers. Retrospective studies, case reports or case series were not included in this search. Participants included patients who had undergone skin grafting, also termed skin transplantation. The intervention of interest was fibrin glue in any form (bovine, human pooled plasma or autologous) and the comparator was any typical form of affixing skin grafts (e.g. sutures or staples). The major outcome of interest was graft survival. Secondary outcomes were rates of haematoma/seroma, postoperative pain, length of stay and operative time.

### 2.3 Information and sources

A search of the English language literature was conducted using the databases MEDLINE, Scopus, Embase, Informit, CINAHL and the Cochrane Central Register of Controlled Trials with no limit on the earliest date of publication. This was last conducted on October 8, 2019.

### 2.4 Search strategy

The aim was to find papers about both fibrin glue and skin grafts or flaps. The first search term of fibrin glue was mapped to the MESH term “Fibrin Tissue Adhesive” and other synonyms such as “Fibrin Glue”, “Fibrin Sealant”, “Tissue Adhesive” and brand names such as “ARTISS”, “Tisseel” and “Beriplast” were included. These terms were all searched using the OR Boolean operator. The second term mapped to the MESH heading of “Skin Transplantation” and synonyms such as “Dermatoplasty” and “Skin Graft” were included using the OR operator. These searches were then combined using the AND operator.

### 2.5 Study selection

Studies were screened by title and abstract and subsequently by full text review for adherence to the eligibility criteria. Studies excluded were those using animal or *in vitro* models and those in which the intervention was not fibrin glue. Abstracts and unpublished studies were included.

### 2.6 Data collection process

Two investigators experienced in systematic reviews reviewed papers for inclusion and extracted data. Any disagreements were referred to a third researcher.

### 2.7 Data items

The eight papers included had data extracted including study design; study duration; sequence generation; allocation; sequence concealment; blinding; other bias; total number of participants; setting; diagnosis; age; sex; country; co-morbidity (in particular vascular comorbidity); total number of intervention groups; intervention (fibrin glue); type of glue (autologous/bovine); alternative intervention (sutures/staples); graft survival; haematoma/seroma/complication; pain; operative time; aesthetic outcome; number of participants in each intervention group; sample size; missing participants; statistical means and standard deviations for outcomes; funding source; study conclusions; miscellaneous comments.

### 2.8 Risk of bias in individual studies

Risk of bias was assessed using the Cochrane risk of bias tool. Bias was assessed in the domains of selection, performance, detection, attrition, reporting and other sources of bias. A funnel plot was planned if ten or more studies were pooled in meta-analysis.

### 2.9 Risk of bias across studies

Publication bias is likely in that studies which did not show any benefit of the fibrin glue may not have been published nor have been registered on a clinical trial database.

### 2.10 Meta-analysis

Meta-analysis was conducted in STATA SE16 [25]. The studies which were meta-analysed were only those using staples or sutures as a comparator as these are the same method of direct fixation around the edge of the graft. Outcomes were reported in literature as mean and standard deviation, number of events over total sample or median and range. Estimates of mean and standard deviation were calculated from medians and ranges as per methodology published in BioMed Central for the purposes of meta-analysis [26]. Authors were contacted for data which was not included in publication which could be used to calculate effect estimates. Further information was provided by authors for two papers [27,28]. A random effects model was used for all cumulative effect calculations. Glass's delta 2 with 95% confidence interval for continuous outcome variables and log odds ratio for binary outcomes were calculated as effect sizes. This was also illustrated in forest plots.

## 3 Results

### 3.1 Study selection

**Fig. 1:**
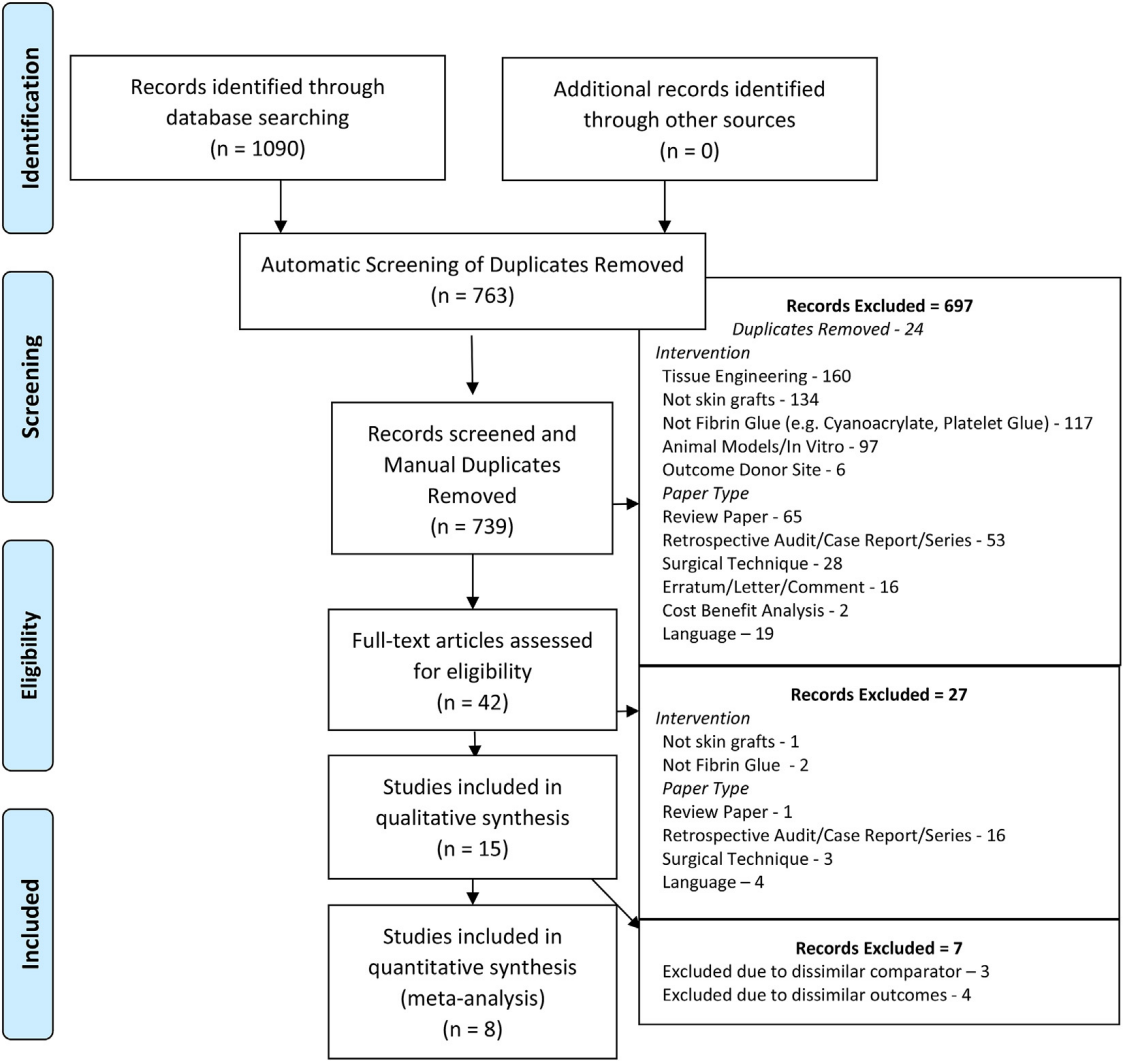
PRISMA flow diagram.

A total of 1090 publications were initially found. Duplicates were removed and 763 records were screened by title and abstract (Fig. 1). Records were excluded if they were not relevant to the topic and common reasons for exclusion were papers studying tissue engineering where the intervention was not the application of fibrin glue, or papers studying animal models of skin grafts. After the initial review, papers were also excluded due to study type (i.e. case reports and review papers). Fifteen studies were found to meet the criteria outlines in the objectives. Twenty-seven papers were excluded based on language, however English translations of abstracts were available for these and none appeared to meet the inclusion criteria.

### 3.2 Study characteristics

Nine of the studies included were randomised controlled trials [[27], [28], [29], [30], [31], [32], [33], [34], [35]], one was a prospective cohort study [36] and the remainder were controlled trials (Table 1a, Table 1b, Table 1ca–c). Studies were published between 1992 and 2019. Only three studies had a total number of participants greater than 50 [28,37,38], and only two studies included skin cancer patients in their population [34,35]. Therefore, 16 of the 594 total patients studied had skin cancer. Many studies included paediatric patients and the overall gender ratio was skewed toward males. The United States of America published five studies on this topic which was more than any other country [[27], [28], [29],^33^,^38^].

**Table 1a:** Summary of paper characteristics.

#	Name	Year	Random Allocation	Total N	N glue	N control	Diagnosis	Age (range: x to x or mean ± SD)	Sex (ratio of Male to Female)	Country
1	Boccara [39]	2017	N	28	28	6	Burns	NI	4.5	France
2	Boeckx [40]	1992	N	27	15	12	Burns	8 to 57	NI	Belgium
3	Burton [29]	2019	Y	17	NI	NI	Burns	39 (Fibrin) 43 (Control)	0.7	USA
4	Dahlstrom [30]	1992	Y	7	7	7	Ulcers	NI	NI	Denmark
5	Danielsen [31]	2008	Y	20	10	10	Ulcers	44 to 86	0.8	Denmark
6	Erba [32]	2010	Y	10	5	5	Ulcers	55 ± 11	9	Switzerland
7	Foster [28]	2008	Y	138	138	138	Burns	1 to 62	1.9	USA
8	Gibran [27]	2007	Y	40	40	40	Burns	6.2 to 54.6	2.6	USA
9	Greenhalgh [33]	1999	Y	47	47	47	Burns	7 to 78	2.9	USA
10	Han [37]	2016	N	55	25	30	Trauma, Burns	NI	NI	South Korea
11	Healy [34]	2013	Y	40	20	20	Trauma, Skin Cancer	60	1.0	UK
12	McGill [38]	1997	N	95	34	61	Burns	10 ± 3.1 (Fibrin) 9.7 ± 3.9 (Control)	1.3	USA
13	Moraes [35]	1998	Y	14	14	14	Skin Cancer	30 to 90	NI	Brazil
14	Reddy [41]	2017	N	16	8	8	Trauma, Burns, Ulcers	13 to 52	1.0	India
15	Youngmin [36]	2018	N	40	20	20	Burns	44 ± 15.2	5.7	South Korea

**Table 1b:** Summary of paper characteristics.

#	Name	Co-morbidity	Type of graft (Split thickness STSG, Full Thickness FTSG)	Type of Fibrin Glue	Control
1	Boccara	NI	STSG	Bovine (ARTISS)	Staples
2	Boeckx	NI	STSG	Bovine (Tisseel)	Sutures (Vicryl)
3	Burton	Excluded	STSG	Not Specified	Staples
4	Dahlstrom	NI	STSG	Autologous	Dressing Only
5	Danielsen	Included	STSG	Autologous (Vivostat)	Staples
6	Erba	NI	Fasciocutaneous Thigh Flap	Bovine (Tisseel)	Sutures (monocryl)
7	Foster	NI	STSG	Bovine (ARTISS)	Staples
8	Gibran	Excluded	STSG	Bovine (ARTISS)	Staples
9	Greenhalgh	NI	STSG	Bovine (ARTISS)	Staples
10	Han	NI	STSG	Human (Greenplast)	Sutures (silk)
11	Healy	Included	STSG	Bovine (Tisseel)	Dressing Only
12	McGill	Excluded	STSG	Human derived fibrin sealant (Baxter)	Staples
13	Moraes	NI	FTSG	Autologous (Glycine Precipitation Technique)	No Grafting
14	Reddy	NI	STSG	Pooled human plasma (EVICEL)	Suture or Staples
15	Youngmin	Excluded	STSG	Human (Greenplast Q; Green Cross Corporation, Yongin, South Korea)	Staples

**Table 1c:** Summary of paper characteristics.

**#**	**Name**	**Funding**	**Graft Survival Related Outcomes**	**Haematoma Seroma**	**Pain**	**LOS**	**Operating Time**	**Other**
1	Boccara	NI	Graft Survival^0^, Healing Time^0^	Y^0^	Y^+^	–	–	Septic Complications^0^
2	Boeckx	NI	–	–	–	–	–	Grip Strength^+^, Key Pinch*, Two Point Discrimination*, Flexion^+^
3	Burton	NI	Graft Loss^0^	–	Y*	Y^0^	–	–
4	Dahlstrom	NI	% Graft Survival^0^	–	–	–	–	Breaking Strength^+^, Bacterial Contamination^0^
5	Danielsen	Funded by Vivolution	Epithelialisation	Y^0^	–	–	–	–
6	Erba	NI	–	–	–	–	–	Flap necrosis^0^, Wound Infection^0^, Drainage time^+^, Drainage volume*
7	Foster	Sponsored by Baxter	Engraftment^+^, Wound Closure*, % Graft Survival^+^	Y*	Y*	–	–	Vancouver Scar Assessment^0^
8	Gibran	Sponsored by Baxter	Assessment of Adherence^+^, Viability*, Graft Survival^0^, Time to Closure^+^, % Graft Survival^0^	Y*	Y^+^	–	–	Wound Size^+^, Pigmentation and Vascularity^0^, Regrafting^0^, Adverse Events^0^
9	Greenhalgh	Sponsored by Baxter and American Red Cross	Healing^+^	–	–	–	–	Viral Safety^0^, Haemostasis*
10	Han	NI	Graft Dislocation^+^, Graft Necrosis*, Graft Survival*	Y*	–	–	–	–
11	Healy	National Institute for Health Research	–	–	Y*	Y^+^	–	Requirement for Dressings^+^
12	McGill	Baxter and the American Red Cross	Time to wound healing^-^	–	–	Y*	Y*	Estimated Blood Loss*
13	Moraes	NI	Wound evaluation^0^	–	–	–	–	–
14	Reddy	Jawaharlal Institute	Wound Closure^0^	Y^+^	–	–	Y^+^	–
15	Youngmin	Korean Health Technology R&D	Graft Survival*	Y^+^	Y*	–	–	Estimated Blood Loss*

^-^ Worse in Fibrin ^0^ No difference+Better in Fibrin * Better in Fibrin (p < 0.05). Underlined outcomes used in meta-analysis.

Only two studies included patients who had vascular comorbidities, one of them being Healy et al. which also included skin cancer patients [31,34]. One study examined fasciocutaenous thigh flaps rather than skin grafts [32], one was full thickness skin grafts [35] with the remainder being split thickness skin grafts. Seven papers examined bovine derived fibrin glue, which was the most common subtype [27,28,[32], [33], [34],^39^,^40^]. Eleven papers compared to sutures or staples for the control group [[27], [28], [29],^31^,^33^,^36^,^39^,^41^]. Five studies were funded by manufacturers of the product studied [27,28,31,33,38].

### 3.3 Meta-analysis

As detailed in Table 1c, the outcomes examined were quite variable in each paper, thus six common outcomes were used for meta-analysis (Fig. 2b, Fig. 2c, Fig. 2d, Fig. 2e, Fig. 2f, Fig. 2aa–f). These are: graft survival by percentage; graft survival reported as events; post-operative incidence of haematoma or seroma; pain reported after dressing changes via a visual analog scale; length of stay in days; and surgical time in minutes. A difference between groups was only seen for length of stay in days (Fig. 2e). A funnel plot was not completed as there was not a sufficient number of studies. Only graft survival reported by event had a heterogeneity less than 50%. All studies included in the meta-analysis posed an intermediate risk of bias, primarily due to randomisation and selection of reported results (Fig. 3).

**Fig. 2a:**
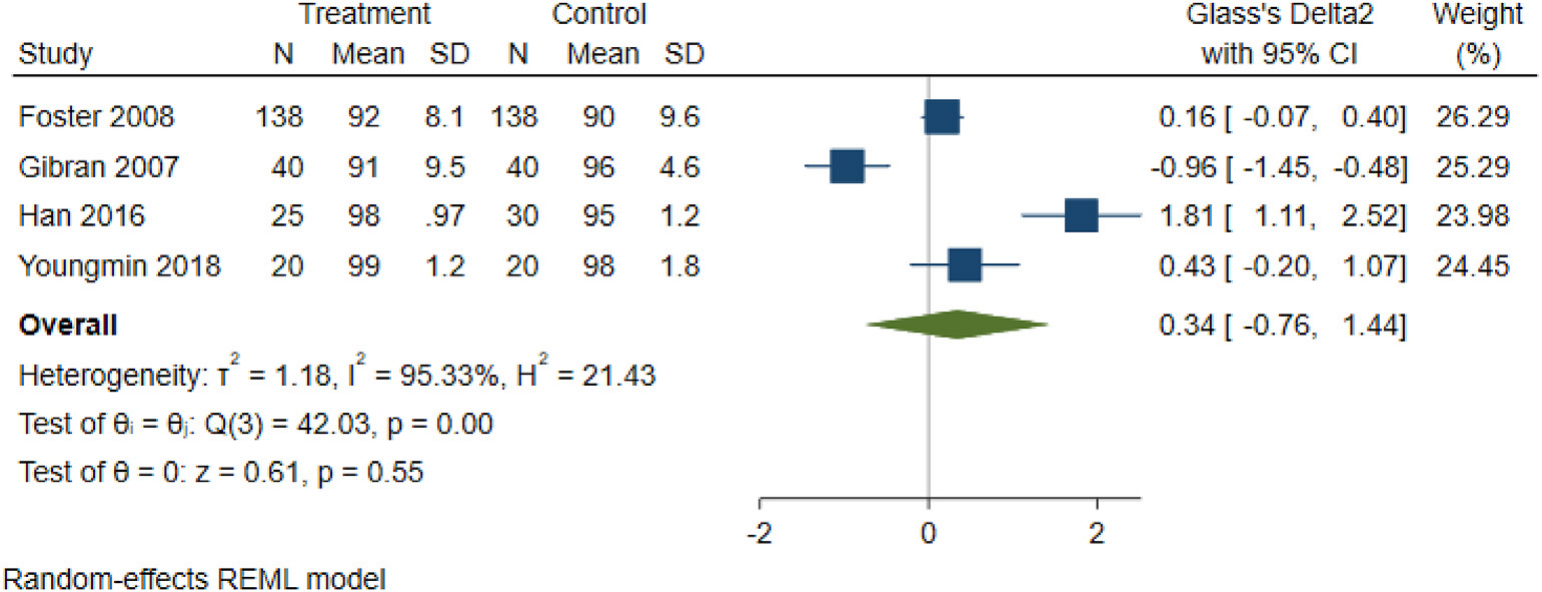
Forest plot of graft survival (%).

**Fig. 2b:**
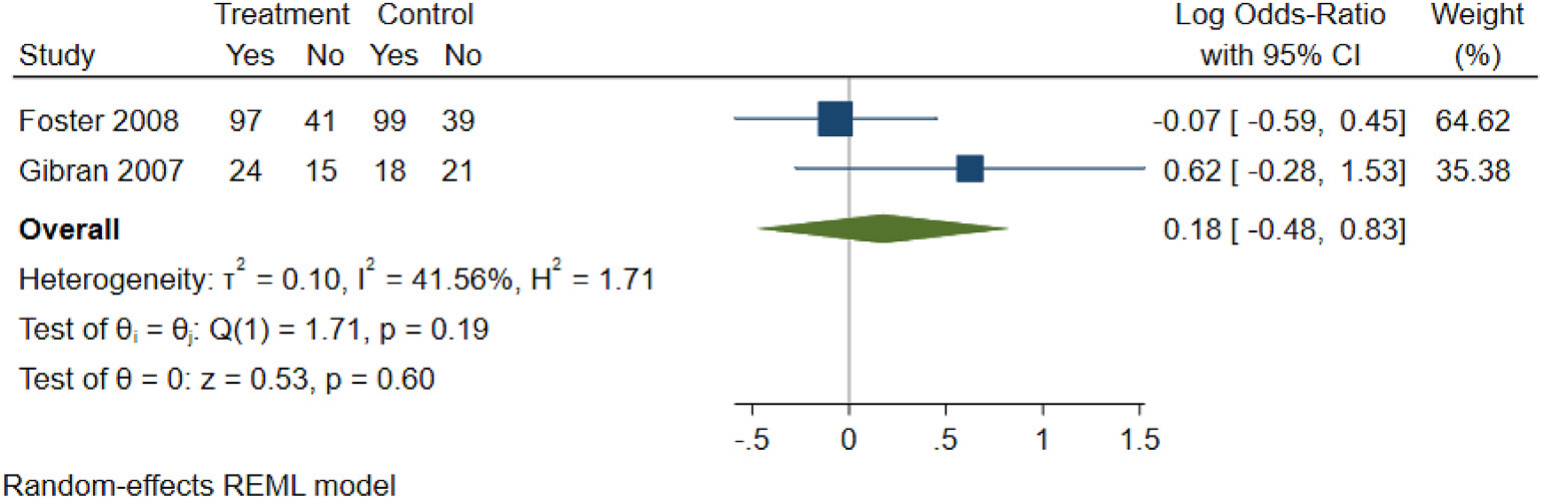
Forest Plot of Graft Survival reported by event.

**Fig. 2c:**
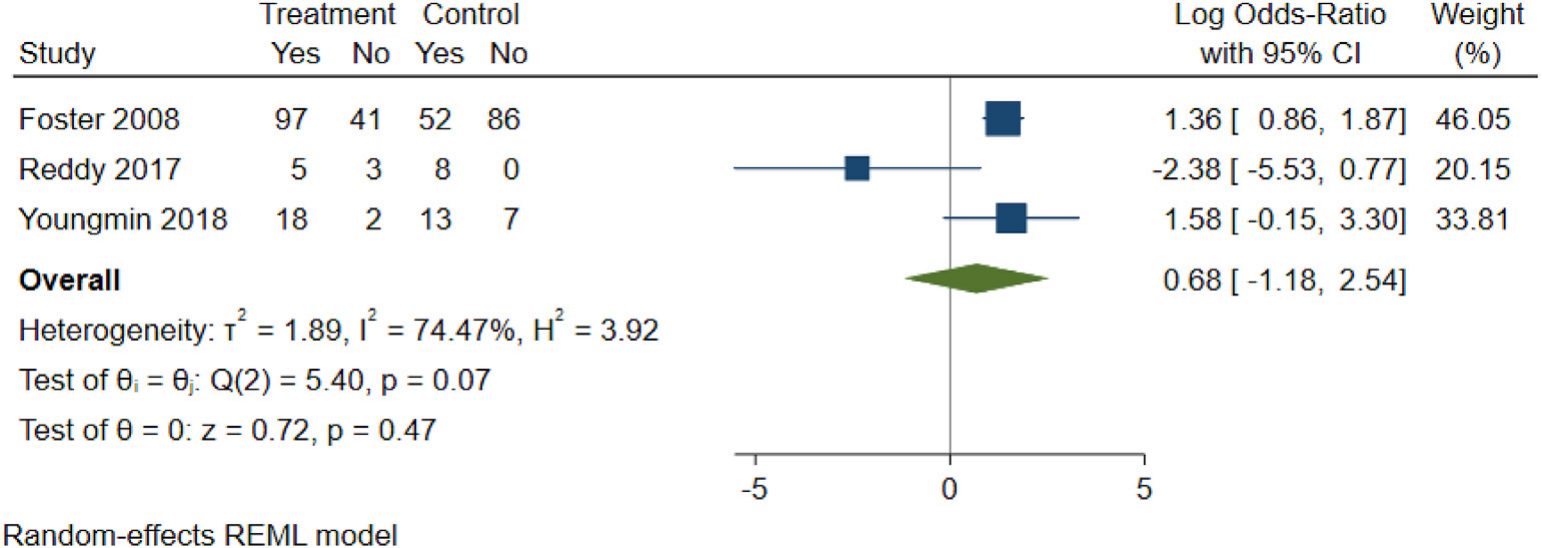
Forest plot of haematoma seroma events.

**Fig. 2d:**
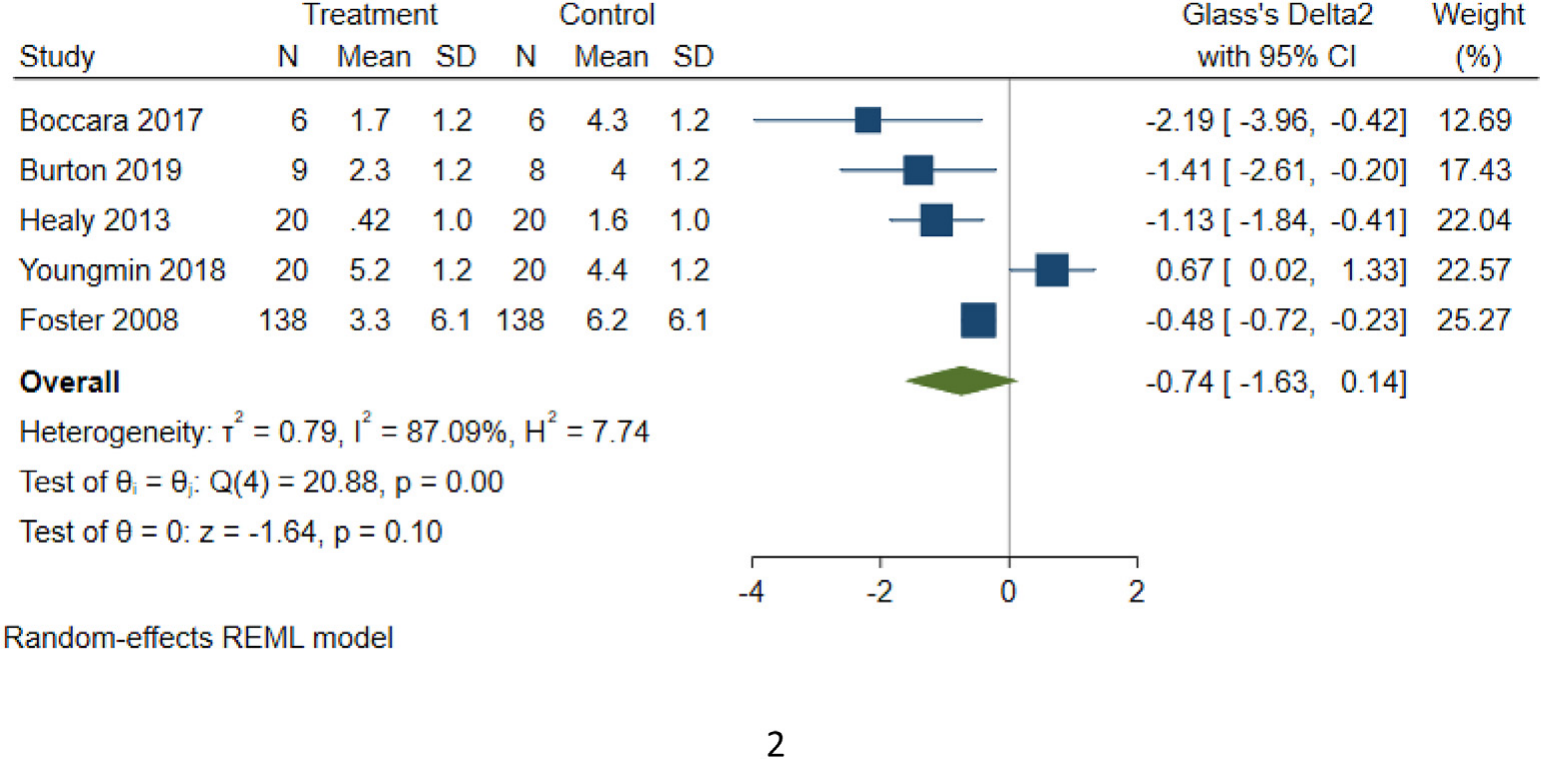
Forest Plot of reported pain after dressing changes (Visual Analog Scale).

**Fig. 2e:**
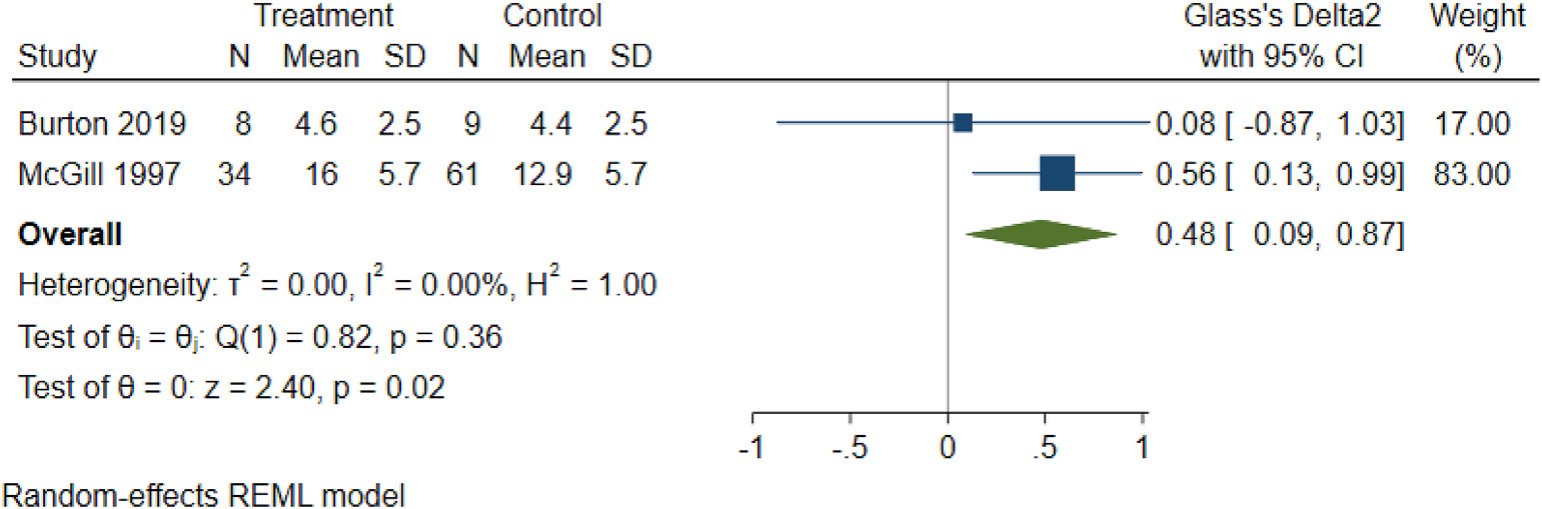
Forest plot of length of stay (Days).

**Fig. 2f:**
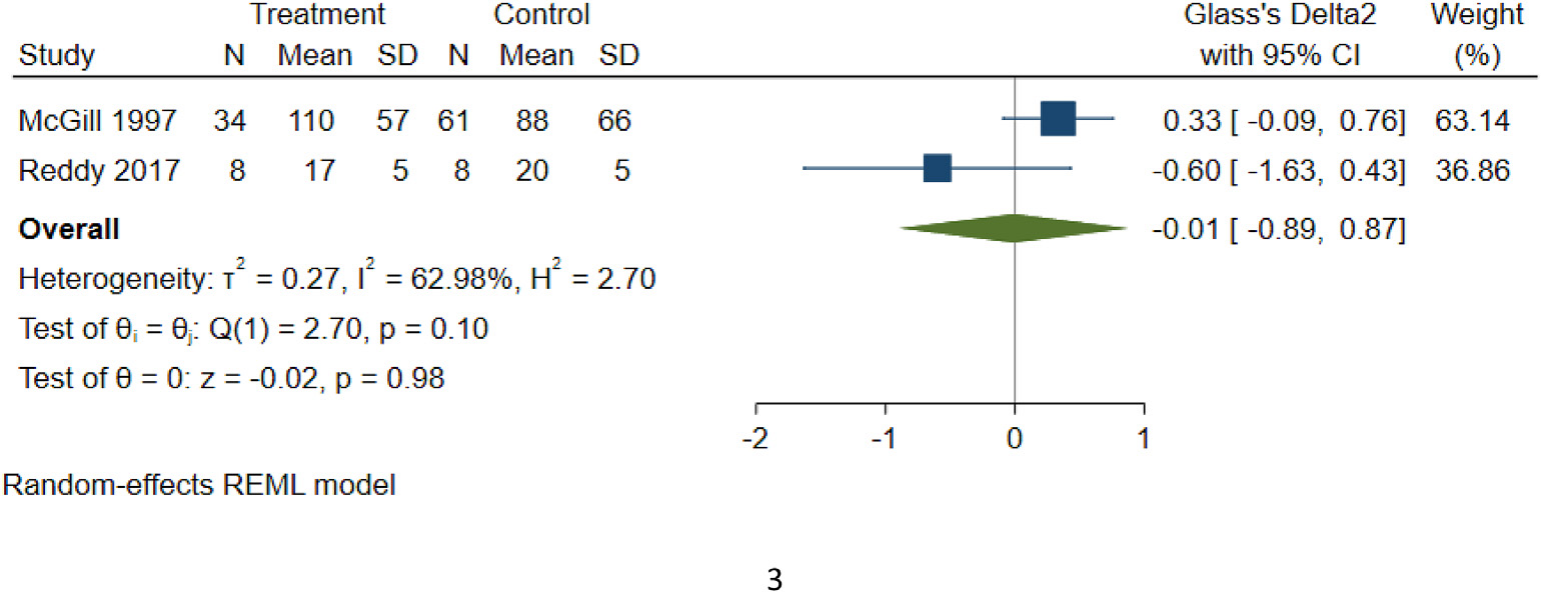
Forest plot of surgery time (minutes).

**Fig. 3:**
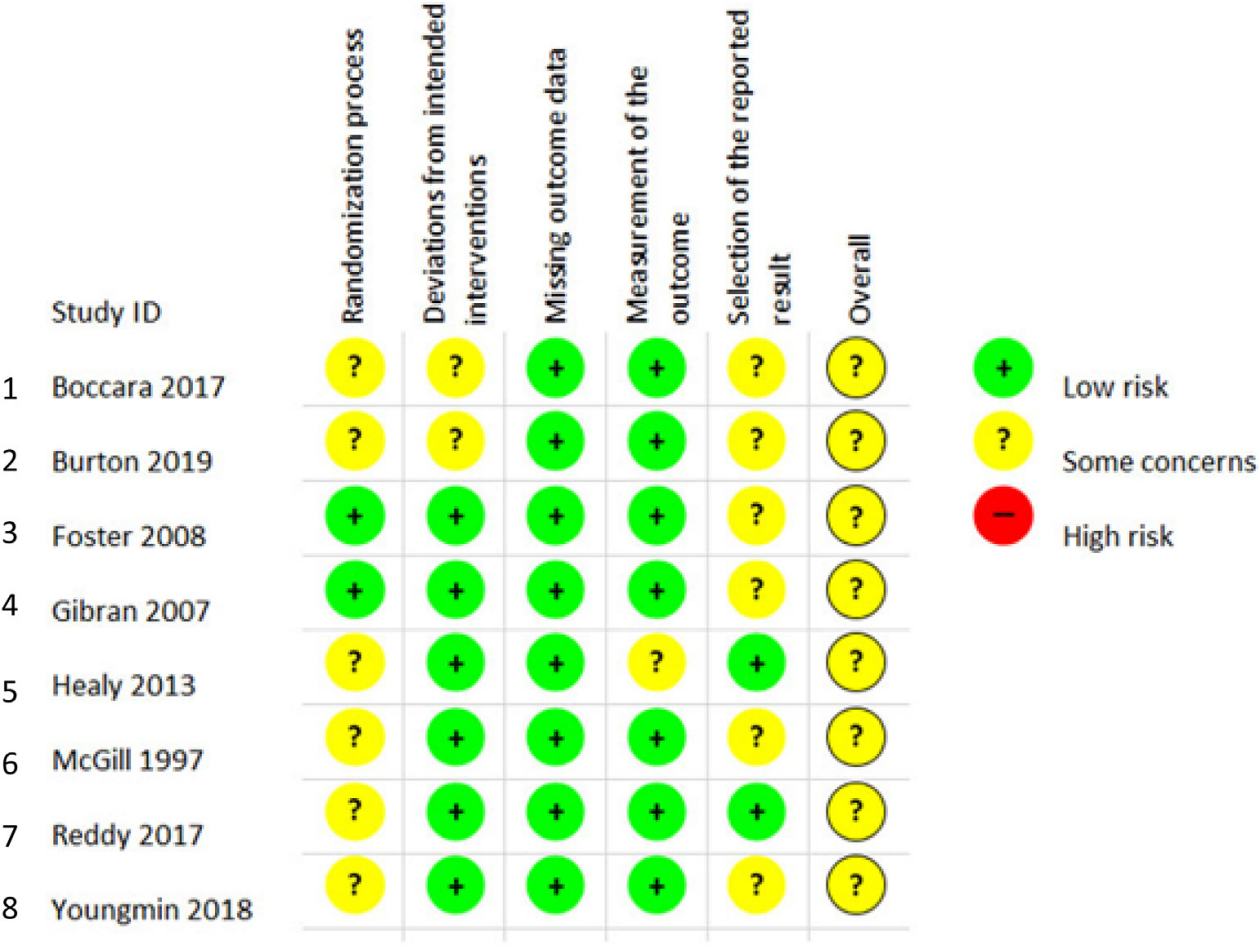
Risk of Bias Assessment for studies included in the meta-analysis.

## 4 Discussion

### 4.1 Summary of evidence

The current published literature does not conclusively demonstrate that fibrin glue is superior to a comparator. The reasons for this might be that fibrin glue is not superior or that this has so far not yet been proven due to only a few small studies available. A large sample size is required since the overall percentage of graft take in both groups being quite high, perhaps due to the exclusion of patients with comorbidities who predispose to lower graft survival, and the existing studies are therefore most likely underpowered.

Most notably, a few papers demonstrated decreased pain after dressing change for patients with fibrin glue used (Fig. 2d). Two articles which did use pain as an endpoint were unable to be included because instead of reporting pain scores, usage of pain relief was recorded [27,28]. Both of these did find that less pain relief was required for patients with fibrin glue [27,28]. Hence, the effect of fibrin glue on pain after dressing change should be explored further.

The other objective of this study was to determine the relevance to the skin cancer population, which frequently require grafts. Unfortunately, there were small numbers of patients with any comorbidity studies, and a paucity of skin cancer grafts. It is difficult to ascertain how relevant these findings may be for this population and dedicated studies should be conducted.

### 4.2 Suitable endpoints

One of the major issues with the current literature is the heterogeneity of endpoints which have been chosen. This has contributed to the risk of bias as there may have been selective reporting of endpoints in studies, for example if haematoma/seroma events were collected but not reported. It also makes comparisons quite difficult as the outcomes have not only been measured differently but reported differently across different studies. For example, one study chose only to look at the effect of skin grafting on mobility and functional outcomes, but no other studies examined these endpoints [40]. This heterogeneity in outcomes may reflect differences in opinions as to which outcomes are important to measure. However, this makes comparison difficult across any studies which examine interventions in skin grafting. Our suggestion would be to establish a pre-determined set of outcomes which reflect clinical measurements, patient reported measures which reflect satisfaction and hospital system measures. Of the outcomes discussed in this paper, graft survival and haematoma/seroma would be clinical; pain, patient reported satisfaction, length of stay and operating time would be hospital system measures. There is a paucity of patient satisfaction related outcomes in the literature, which is important to consider in skin grafting where the outcomes are immediately apparent.

### 4.3 Limitations

Bias and the quality of the published literature were limiting factors in this review. Funding from commercial companies is of concern, although most papers noted that company representatives were not involved in the study design or implementation. Poor randomisation methods such as lack of allocation concealment also contributed to issues in many studies. Blinding is difficult with this intervention as it is immediately apparent to patients and carers as soon as dressings are removed whether staples or fibrin glue were used. Some studies had independent assessors or photographic software analysis to attempt to overcome this [28,36]. Furthermore, early studies were not required to undergo trial registry and so protocols were not accessible. Authors were contacted for extra information, but this was not always successful. A final limitation is that non-English papers were excluded.

## 5 Conclusions

While there may be benefits to the use of fibrin glue in skin graft patients, it is difficult to conclude this from the current evidence. The papers published focus on many different outcomes, and as a recommendation we would suggest some standard outcomes are used in future skin graft research. In addition, further high quality randomised controlled trials with large comparison groups are necessary to determine the usefulness of fibrin glue in clinical practice.

## Funding statement

The authors received no financial support for the research, authorship, and publication of this article.

## Provenance and peer review

Not commissioned, externally peer reviewed.
